# Assessment of cerebral autoregulation indices – a modelling perspective

**DOI:** 10.1038/s41598-020-66346-6

**Published:** 2020-06-15

**Authors:** Xiuyun Liu, Marek Czosnyka, Joseph Donnelly, Danilo Cardim, Manuel Cabeleira, Despina Aphroditi Lalou, Xiao Hu, Peter J. Hutchinson, Peter Smielewski

**Affiliations:** 1 Brain Physics Laboratory, Division of Neurosurgery, Department of Clinical Neurosciences, Addenbrooke’s Hospital, University of Cambridge, Cambridge, UK; 2 Department of Anesthesiology & Critical Care Medicine, School of Medicine, Johns Hopkins University, Baltimore, MD USA; 3 Institute of Electronic Systems, Warsaw University of Technology, Warszawa, Poland; 4 Department of Anaesthesiology, University of Auckland, Auckland, New Zealand; 5 Department of Neurology and Neurotherapeutics, University of Texas Southwestern Medical Center, Dallas, USA; 6 School of Nursing, Duke University, Durham, NC USA

**Keywords:** Experimental models of disease, Dynamical systems, Neurology

## Abstract

Various methodologies to assess cerebral autoregulation (CA) have been developed, including model - based methods (e.g. autoregulation index, ARI), correlation coefficient - based methods (e.g. mean flow index, Mx), and frequency domain - based methods (e.g. transfer function analysis, TF). Our understanding of relationships among CA indices remains limited, partly due to disagreement of different studies by using real physiological signals, which introduce confounding factors. The influence of exogenous noise on CA parameters needs further investigation. Using a set of artificial cerebral blood flow velocities (CBFV) generated from a well-known CA model, this study aims to cross-validate the relationship among CA indices in a more controlled environment. Real arterial blood pressure (ABP) measurements from 34 traumatic brain injury patients were applied to create artificial CBFVs. Each ABP recording was used to create 10 CBFVs corresponding to 10 CA levels (ARI from 0 to 9). Mx, TF phase, gain and coherence in low frequency (LF) and very low frequency (VLF) were calculated. The influence of exogenous noise was investigated by adding three levels of colored noise to the artificial CBFVs. The result showed a significant negative relationship between Mx and ARI (r = −0.95, p < 0.001), and it became almost purely linear when ARI is between 3 to 6. For transfer function parameters, ARI positively related with phase (r = 0.99 at VLF and 0.93 at LF, p < 0.001) and negatively related with gain_VLF(r = −0.98, p < 0.001). Exogenous noise changed the actual values of the CA parameters and increased the standard deviation. Our results show that different methods can lead to poor correlation between some of the autoregulation parameters even under well controlled situations, undisturbed by unknown confounding factors. They also highlighted the importance of exogenous noise, showing that even the same CA value might correspond to different CA levels under different ‘noise’ conditions.

## Introduction

Cerebral autoregulation (CA) refers to the active control of cerebral resistive arterioles in response to increased or decreased cerebral perfusion pressure (CPP) or arterial blood pressure (ABP), and is an important homeostatic mechanism that protects the brain against injury due to potentially insufficient or excessive cerebral blood flow (CBF)^1–6^.

In the last 20 years, a wide variety of techniques have been developed and adopted for CA assessment, include the autoregulation index (ARI), transfer function analysis (TF, including phase shift, gain and coherence), mean flow index (Mx), etc^7–15^. Despite advances in the application of CA assessment^16,17^, there still remains no consensus on which approach can be considered as ‘gold standard’^6,18,19^. Although a few comparisons between various CA parameters have been published ^20–27^ based on real ABP and cerebral blood flow velocity (CBFV) measurements, testing the integrity of CA remains a major technical challenge^2^. For example, some studies showed that TF parameters correlated with ARI, while other studies found no relationship between the two; some studies demonstrated negative relationship between TF gain and phase, while several studies showed fairly weak strength of correlations between them^8,26,28,29^. Other investigators have also characterized CA using several metrics, leading to different outcomes^30–33^. In most recent work, Sanders *et al*. reported pool reproducibility in ARI and correlation method using data from 14 centers^34^. Whether the poor convergence between these CA metrics is due to fundamental differences of various algorithmic models or is caused by unknown extraneous ‘noise’^14^ presented in the real data (i.e. components in ABP and CBFV which are not related to each other), needs further studying. Moreover, unknown noise makes meaningful comparisons among various CA methods difficult and further investigations about the influence of noise need to be done.

This pilot study aims to assess the relationship among three commonly used CA indices in a more controlled environment and assess the influence of noise on CA assessment^35^. Artificial CBFV signals were generated according to Tiecks’ ARI model^1^ by using real ABP signals as the input. Mx and TF parameters were calculated and compared with ARI values based on well controlled situations, undisturbed by unknown confounding factors. Stimulated by Panerai *et al*.^36^, a varying degree of ‘exogenous’ noise was imposed on the simulated data to estimate the influence of noise on these relationships. The analysis mainly focuses on two frequency ranges following the recommendations by the International Cerebral Autoregulation Research Network (CARNet) white paper^37,38^: very low frequency range (VLF, 0.02~ 0.07 Hz) and low frequency range (LF, 0.07~ 0.2 Hz). We use gain_VLF for the abbreviation of gain in VLF range, and gain_LF for gain in LF. Similarly, phase_VLF and phase_LF refer to phase in the VLF and LF range, while coh_VLF and coh_LF stand for squared coherence in the VLF and LF range respectively. For technical details, please refer to the methodology section at the end of the paper.

## Results

### Simulated CBFV

**Figure 1:**
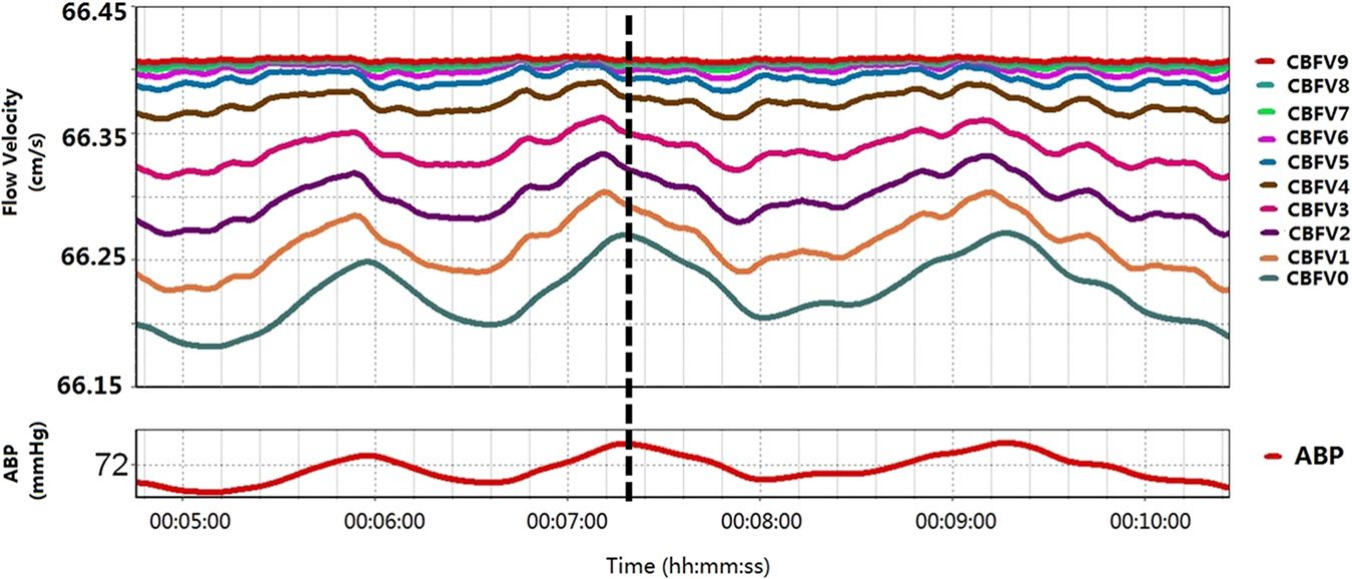
An example of ten artificial flow velocities (upper panel) created according to the real mean ABP (lower panel) using Tiecks’ ARI model. CBFV0 refers to the CBFV generated according to the model using dysfunctional autoregulation indices (ARI 0). CBFV9 refers to the flow velocity generated according to the model of hyperactive autoregulation (ARI 9). In order to show the phase shift much clearer, the CBFVs shown in the picture has been filtered using a moving average filter of 45 s window. ARI: autoregulation index.

The average age of this cohort was 28.8 years (standard deviation, SD, 15.9 years) with 8 females and 26 males. Mean ABP was 82.0 ± 10.5 mm Hg (mean ± SD). One example of 10 artificially generated CBFVs (upper panel) from a fragment of a real ABP signal recording (lower panel) is shown in Fig. 1. In order to show the phase shift between different CBFVs and ABP more clearly, a moving average filter of 45 s window was applied to the artificial CBFVs in Fig. 1. Differences in amplitudes and phases of the ten CBFVs are clearly visible. To clarify, the 45-second moving average window was only applied in Fig. 1 to improve the visualization. In the following calculations of CA parameters, this filter was not used. This is because in Fig. 1, the original artificial signal created by Tickes’ model were used, which still contain the high frequency components (pulse and respiratory waves). Therefore in order to visualize differences in phase shift and gain of the generated CBFV signals, we have to strip the high frequency components by low pass filtering (the 45 sec moving average filter, to filter out the components above 0.2 Hz).

### Relationship between ARI and Mx under no-noise condition

**Figure 2:**
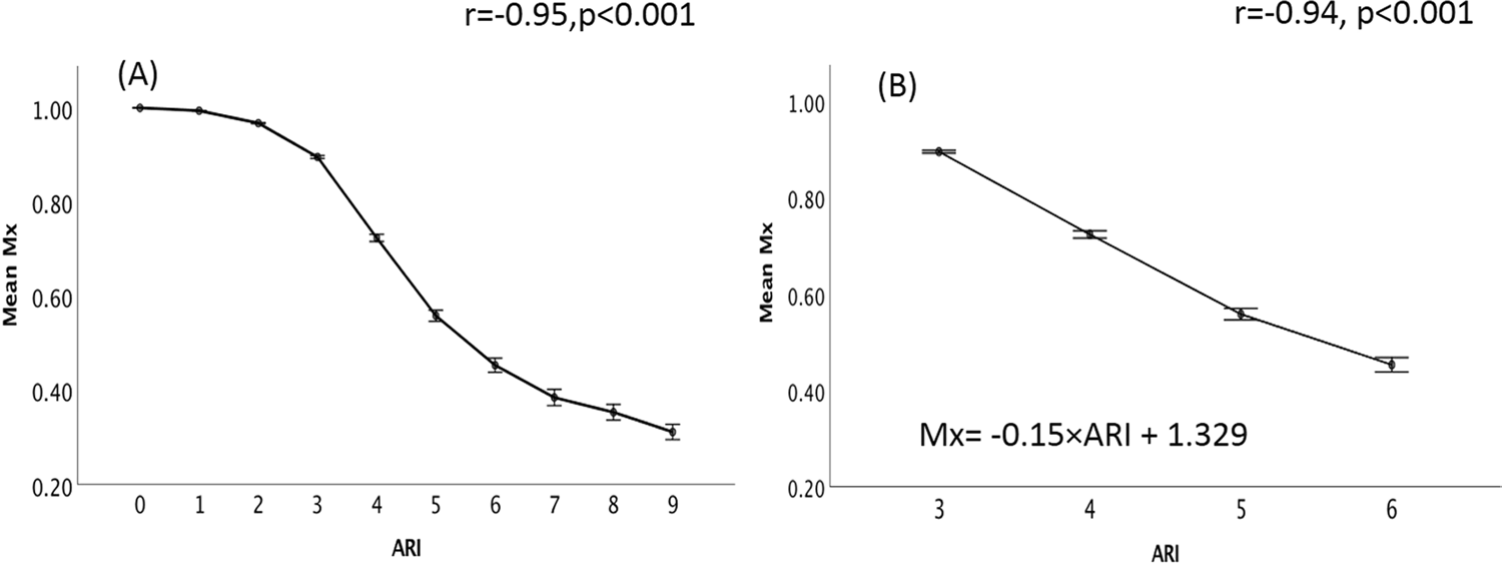
The relationship between mean Mx and ARI under no-noise condition. (**A**) Mean value of Mx for different groups of artificial flow velocities generated from ARI0 to ARI9. (**B**) Mean value of Mx from ARI3 to ARI6, we can see a linear relationship between these two parameters. Mx: mean flow index using arterial blood pressure as input; ARI: autoregulation index. Error bar: standard error.

Mx negatively correlates with ARI (r = −0.95, p < 0.001, n = 34, Fig. 2A). From ARI 3 to ARI 6, the usual range of values seen in clinical practice, the relationship between these two indexes can be described as: Mx = −0.15×ARI + 1.329 (Fig. 2B, r = −0.94, n = 34, p < 0.001).

### Relationship between ARI and estimated TF parameters under no-noise condition

**Figure 3:**
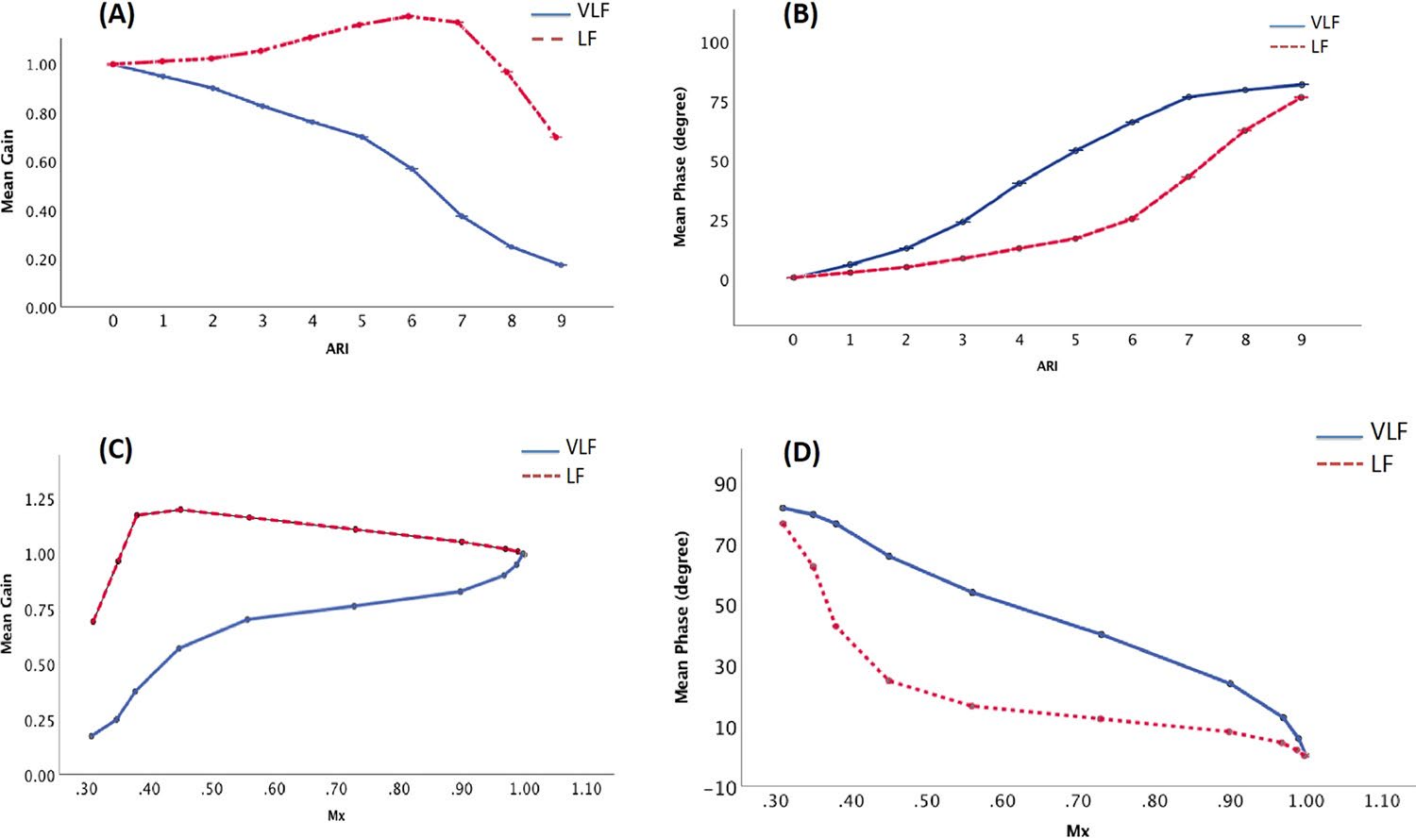
(**A**,**B**) The relationship between ARI and the estimated transfer function parameters under no-noise condition. (**C**,**D**) Relationship between Mx with transfer function parameters under no-noise condition. ARI: autoregulation index; Mx: mean flow index; VLF: very low frequency range, 0.02–0.07 Hz; LF: low frequency range, 0.07-0.2 Hz. Gain_VLF: transfer function gain in VLF; Gain_LF: gain in LF; Phase_VLF: phase in VLF; Phase_LF: phase in LF.

Figure 3A, B describes the relationships between ARI and TF parameters in both VLF and LF ranges. There is a significantly negative relationship between ARI and Gain_VLF (Fig. 3A, r = −0.98, p < 0.001). In both frequency ranges, there is a positive and highly monotonic relationship between phase and ARI, with r = 0.99 (p < 0.001) at VLF and r = 0.93 at LF (p < 0.001). Furthermore, it becomes almost purely linear when ARI is between 3 to 6 at VLF (phase = 13.97 × ARI – 17.06, r = 0.998, p < 0.001, Fig. 3B).

### Relationship between Mx and TF parameters under no-noise condition

A nearly linear, negative relationship exists between Mx and Phase_VLF (Phase_VLF = 117.4 – 110.6 × Mx; r = −0.97, p < 0.001, Fig. 3D), which is not quite the case for Mx and phase_LF (r = −0.86, p < 0.001, Fig. 3D). Gain at VLF was also monotonically related to Mx (r = 0.93, p < 0.001, Fig. 3C), while gain at LF did not show a monotonous relationship with Mx (p = 0.066, Fig. 3C).

### Relationship among TF parameters under no-noise condition

This cohort of simulated data shows a strong relationship among transfer function gain and phase. TF gain negatively correlates with phase both in VLF and LF (r = −0.96, p < 0.001 between gain_VLF and phase_VLF, r = −0.52, p < 0.001 between gain_LF and phase_LF). Coherence showed significant relationship with gain and phase at VLF (r = 0.82, p < 0.001 between gain_VLF and coh_VLF, r = −0.90, p < 0.001 between phase_VLF and coh_VLF). However, the relationship between coherence and gain in LF is extremely weak (r = 0.13, p = 0.016) as well as coherence and phase in LF (r = −0.13, p = 0.020).

### Analysis of the effects of exogenous noise

**Figure 4:**
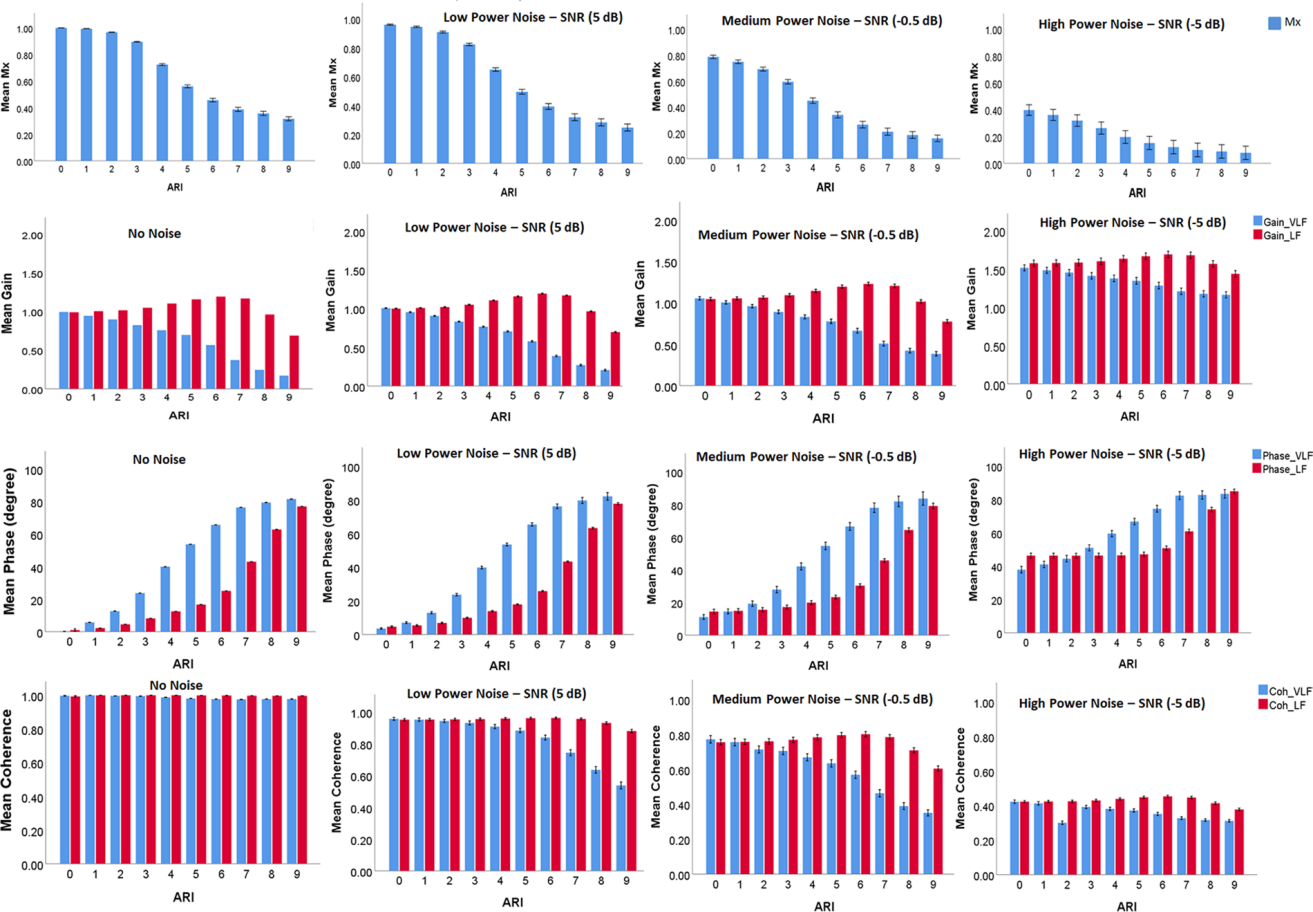
The relationships between Mx, TF parameters and ARI with different intensities of added noise. In the panel grid rows (from up to bottom) represent different indices: Mx, Gain, Phase and Coherence, and columns (from left to right) represent increasing intensity of noise. SNR: signal to noise ratio; Mx: mean flow index; ARI: autoregulation index; TF: transfer function. Error bar: standard error.

Figure 4 displays the relationship between ARI and other CA parameters using CBFV without noise as well as CBFV with artificial noise at three signal-to-noise ratio (SNR) levels: high SNR (5 dB), medium SNR (−0.5 dB) and high SNR (−5 dB) power noise. Despite a general preservation of the overall character of the relationship between Mx and ARI, the actual value changes with the different levels of noise. With increased noise, Gain was increased, and coherence was decreased in general. The character of the relationship between TF phase and ARI was kept almost the same. We also tested the relationship between estimated TF gain and ARI under different levels of artificial noise (Table 1).

**Table 1:** The Pearson’s correlation coefficient (r) between ARI and other cerebral autoregulation parameters.

	No Noise	Low Noise	Middle Noise	High Noise
Mx	r = −0.95, p < 0.001	r = −0.93, p < 0.001	r = −0.87, p < 0.001	r = −0.71, p < 0.001
Gain_VLF	r = −0.98, p < 0.001	r = −0.98, p < 0.001	r = −0.94, p < 0.001	r = −0.45, p < 0.001
Gain_LF	r = −0.22, p < 0.001	r = −0.23, p < 0.001	r = −0.20, p < 0.001	p = 0.49
Phase_VLF	r = 0.99, p < 0.001	r = 0.98, p < 0.001	r = 0.95, p < 0.001	r = 0.81, p < 0.001
Phase_LF	r = 0.93, p < 0.001	r = 0.92, p < 0.001	r = 0.88, p < 0.001	r = 0.71, p < 0.001
Coh_VLF	r = −0.86, p < 0.001	r = −0.87, p < 0.001	r = −0.90, p < 0.001	r = −0.44, p < 0.001
Coh_LF	p = 0.73	r = −0.42, p < 0.001	r = −0.36, p < 0.001	r = −0.11, p=0.04

ARI: autoregulation index. TF: transfer function; Mx: mean flow index using arterial blood pressure as input; VLF: very low frequency, 0.02~ 0.07 Hz; LF: low frequency, 0.07~ 0.2 Hz. Black: TF parameters in very low frequency; p value for all the parameters in this form is below 0.01. Gain_VLF, Phase_VLF, Coh_VLF refers to gain, phase and squared coherence in VLF range; Gain_LF, Phase_LF, Coh_LF refers to gain, phase and squared coherence in LF range. P < 0.05 was considered to be significant.

**Figure 5:**
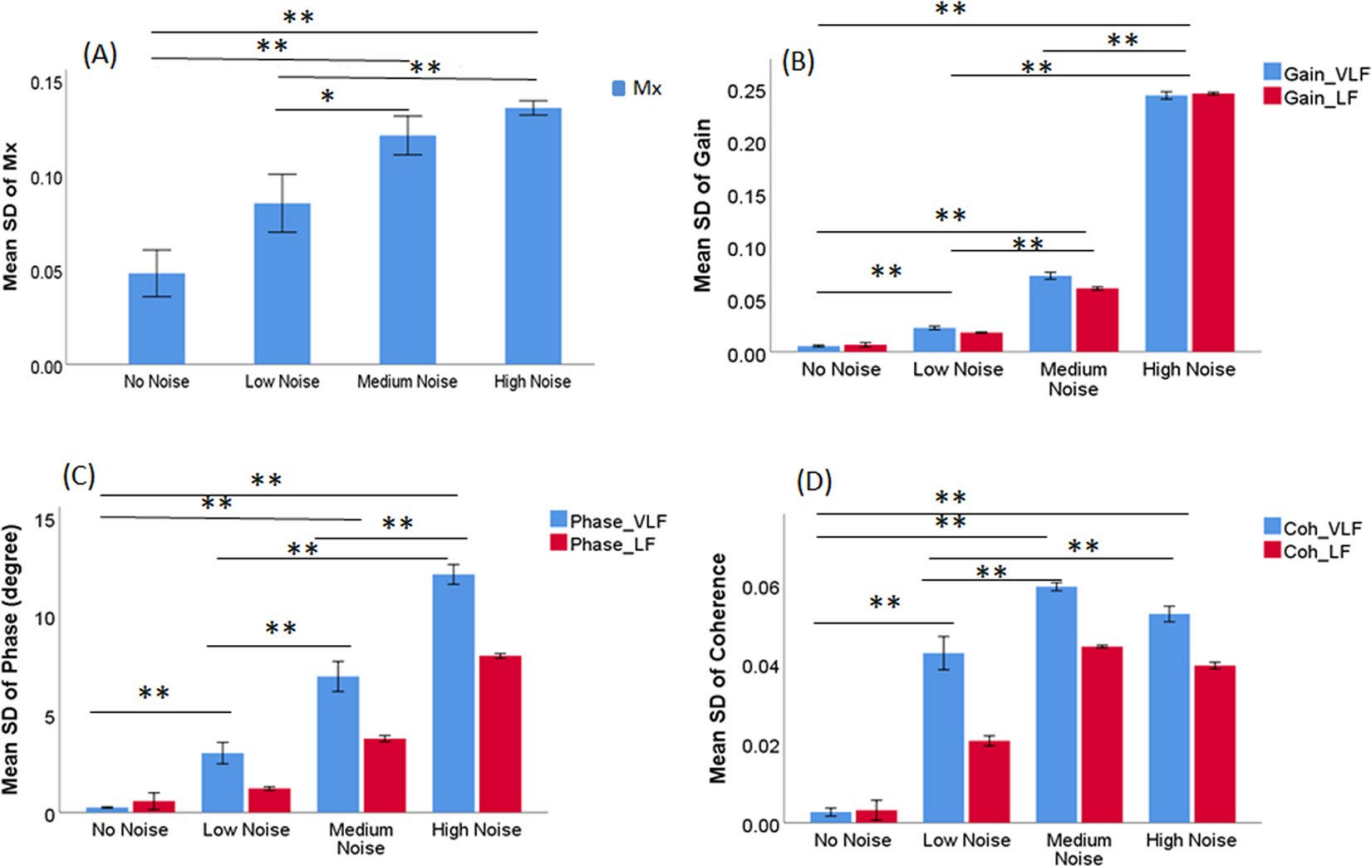
Mean standard deviation of Mx (**A**), transfer function gain (**B**), phase (**C**) and coherence (**D**) at different nose levels. SNR: signal to noise ratio; Mx: mean flow index; VLF: very low frequency; LF: low frequency. ** indicates p value was smaller than 0.001, and * indicates p value was smaller than 0.05.

Figure 5 shows that, as expected, the standard deviations of the studied parameters were increased with added noise, especially while high power noise was added. Furthermore, in the presence of noise, a monotonic relationship between the estimated coherence and ARI was revealed in the VLF range (Fig. 4).

## Discussion

In the past two decades, quite a few studies analyzed the relationships among CA parameters in different circumstances^8,26,28–32^, however, the results vary. Whether the poor convergence between CA metrics is due to fundamental differences of various algorithmic models or is caused by unknown extraneous ‘noise’^14^ presented in the real data needs further studying. In their study, Simpson and his colleagues confirmed that even within a given recording of the same patient, ARI estimates can be inconsistent^6^, probably due to short-term variations in autoregulatory activity, nonlinear system characteristics^39^, as well as the influence of other physiological variables (e.g., CO2 and O2 levels and intracranial pressure variations^40^) on CBFV. In order to exclude these uncertain elements in CA calculations, this study explored the interrelationships among three commonly used CA metrics: 1) transfer function analysis, 2) a second-order linear model (ARI), and 3) time-based correlation (Mx), by using a cohort of artificial CBFVs. We demonstrate strong but non-linear relationships between ARI and Mx, ARI and phase as well as between phase and Mx. In addition, our study also showed that the intensity of noise had a profound influence on all CA parameters. These analyses add insight to both the interpretation of our previously published literature and the design of future cerebral haemodynamics investigations.

Using the simulated CBFVs, we artificially made ‘ARI’ as a reference to compare with other method. The character of the relationship between Mx and ARI confirmed the metric convergence and general interchangeability of the two indices from a mathematical level. However, considering the shape of the relationship follows an inverted ‘S’ - shaped curve (Fig. 2), Mx may only be used to grade CA levels in the middle range (corresponding to ARI 3 to 6), beyond which the shape saturates. For noise-free simulation, the range that Mx can be used as a scale is between 0.38 to 0.90 (corresponding to ARI 3 to 6). However, in practice, as shown by our noise simulations, one should expect this range to be shorter and shifted down towards lower values of Mx (Fig. 4A).

A linear system is always a priori assumption while using TF for CA assessment. This cohort of artificial data met the criteria, and the consistent relationships among TF phase with ARI or Mx supported the theoretical interchangeability of these metrics. In terms of grading different CA levels, the roughly ‘S’ - shaped relationship between phase and ARI indicates that for a noise-free situation, a phase from 0 to 70 degrees can be used to grade CA (corresponding to ARI 1 to 7, Fig. 3B). With ‘noise’ added, the relationship is slightly flattened (Fig. 4). Gain in the LF band did not show a monotonous relationship with either ARI or Mx, and therefore its use in LF for CA analysis cannot be recommended. This might be explained by the pre-processing procedure: normalization. In this study, the ABP and CBFV were normalized into Z scores (mean subtracted, and divided by the standard deviation) prior to TF analysis. According to the white paper (recommendation 8)^37^, removing mean values prior to TF analysis is useful to minimise spectral leakage. However, arguments against normalizing ABP (or CBFV) as % (relative to the mean) rather than in absolute units (mmHg or cm/s) have been raised. Since a 10% change for example, would be physiologically very distinct for a patient with a baseline mean ABP of 90 mmHg, compared to an individual with a baseline mean of 150 mmHg^37^. The normalization by the mean value would reduce intersubject variability of CBFV amplitude and also affect the gain estimates directly^37^, thus influencing the shape of the relationship between Gain and ARI. This maybe the main reason why LF gain comes out so poorly (poor correlation with ARI) in the analysis of this paper and it also tells us that we need to be very careful while we use gain for CA assessment. Therefore more studies to investigate the effect of normalization are needed.

In this study, the TF phase was unwrapped in degrees to [−180°, 180°]. As mentioned in the white paper^37^, for phase unwrapping, distorted estimates of mean phase will result from averaging positive and negative values while the latter results from phase ‘wrap-around’ in the VLF or LF frequency bands. Therefore, in this study we upwrapped the phase to [−180°, 180^0^] degrees and the negative values was removed for later analysis following the guidelines in the white paper^37^.

Previous studies using real data showed conflicting results on the relationships among CA parameters^8,26,28^. This disagreement might be due to various reasons: 1) different basic analytic constructs; therefore they may not reflect the same aspect of the underlying physiological response^4^; 2) unrelated and unknown noise in the real data that influence the results;^34,41–43^ 3) low reproducibility of CA parameters that might differ in different groups of subjects^30,32^, or 4) inappropriate hypothesis, for example the TF analysis assumes CA as a stationary system, while this is not true in reality^27,44^. This study shows that different methods can lead to poor correlation between some of the autoregulation parameters (e.g. the non-significant ones found here) even in well controlled simulated data, with a simple linear model. This provides an additional explanation for why clinical measures can be poorly correlated.

**Figure 6:**
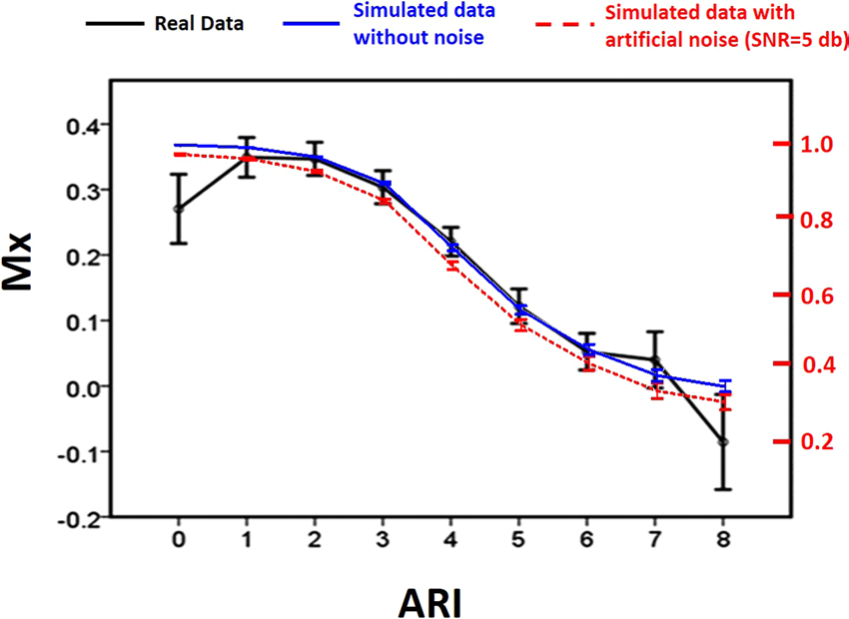
Comparison of Mx-ARI relationship between real data and modelled data. The real curve measured in a cohort of TBI patients (solid black) is plotted against curve obtained using modelled data under low-power noise conditions (red dotted line), and against modelled data without noise (solid blue line). The Mx value of real data were shown on left y axis; and the Mx value of modelled data were shown on right y axis. SNR: signal to noise ratio; ARI: autoregulation index; Mx: Mean flow index.

By generating artificial CBFV data, the original analyses necessarily excluded all external noise from the CA estimates. In order to simulate a more realistic scenario and investigate the influences of the noise on CA assessment, we used three levels of intensity of additive artificial noise (SNR: 5 dB, −0.5 dB, −5 dB). Katsogridakis *et al*. found that the distribution of SNR of real CBFV measurement was mainly between 4–6 dB in a study of 60 volunteers^45^. Therefore, we chose artificial noise of SNR = 5 dB to approximates the real-world scenario of clinical CA assessment. The comparison with previously published data indicates that such artificial noise may be relevant^29^. The relationship between ARI and Mx using the simulated data with artificial noise in the current analysis is qualitatively similar to the relationship between ARI and Mx derived from our previous study using real data of 288 TBI patients (Fig. 6)^29^. This reasonably good match indicates that our rather simplistic approach might provide an acceptable approximation to the exogenous noise seen in real data.

As expected, the intensity of noise has a significant influence on all CA parameters, particularly on the relationship between (TF) coherence and ARI. With the linear model used in this work, in the absence of noise, one would expect coherence to be high (almost 1 in the estimates), regardless of ARI (the coherence does not care about what the relationship between input and output is, just that the relationship is strong and linear - as is the case for these filters and no noise). However, with different noise intensities, the character of the coherence-ARI relationship changes dramatically. Coherence is useful in detecting strongly non-linear relationships as expected when autoregulation is strong (but not modelled with the ARI filters) or noise in the data. The other TF parameters (i.e. phase and gain) together with Mx, and their relationships to ARI were affected by the noise to various extents. Interestingly, although the shape of the relationship curve between the parameters and ARI remained largely unchanged, the scaling was significantly affected. With increasing noise, Mx tends to decrease due to unrelated components introduced by noise. As small Mx refers to good autoregulation, with increasing noise, Mx would overestimate autoregulation (Mx tends to be low). Therefore, in different ‘noise’ conditions (any exogenous process), the same TF parameter value or Mx value will correspond to different autoregulation (i.e. strengths of CA) levels. This can potentially explain the poor reproducibility of CA parameters across different patient cohorts.

Recalibration of the various CA indices according to the ‘background’ exogenous ‘noise’ for a particular patient population may therefore be necessary for consistent CA grading. This may of course not be a practical solution, as the level of ‘background noise’ will be in reality unknown, possibly variable and generally unmeasurable. Katsogridakis *et al*. provided a reasonable way for noise calculation^45^. If one, for example, compares the real Mx-ARI characteristics measured in TBI patients^29^, with a theoretical Mx-ARI relationship curve using the simulated data (Fig. 6), the two curves have similar shapes and match well with each other.

## Limitations

In this study we used a time-invariant model, a second-order linear model (Tiecks’ model), to generate CBFV signals, and it assumes the relationship between input and output to be linear. While in reality, the impact of many other variables and confounding factors (such as CO2), make the relationship complex. We need to bear in mind that the conclusion about the relationship between the CA parameters in this study is predominantly held for the artificial data and is from a mathematical point of view. The result may not accurately reflect the actual relationships of realistic recordings, as discussed by Panerai *et al*.^44^. It is important to note that the confounding effects of noise on these CA parameters might be helpful, but the relationship between the parameters using simulated data with no noise is too ideal and can not be considered to be true in real situation. Moreover, the data we used for modeling was taken from TBI patients, other cohorts of patients need to be tested for further studies.

Moreover, we fully acknowledge that our representation of confounding exogenous processes and non-linear effects as an additive colored Gaussian noise signal is a gross simplification. As we have no clear grounds to shape the noise to simulate the real situation, due to the reason that the spectral distribution of the real noise varies between individual recordings, we have to choose the most generic type of noise assuming that it will highlight the effects of noise on CA parameters. Although the noise simulation provided us with a possible way of investigating the effects of noise on the performance and interrelationships of these CA indices, we did not take individual differences of noise into account. Finally, given that the SNR was calculated over the whole signal frequency band, but then the noise was filtered, removing much of its power, the true (relevant) SNR for the signal (frequency band of interest) is much higher.

## Conclusions

This study explored the relationships between the most commonly used indices of CA: ARI, Mx and TF parameters under well controlled situations, undisturbed by unknown confounding factors. The results show that under no-noise condition, ARI, Mx and TF phase (but not gain) were interchangeable while ARI is between 3 to 6. Coherence should be only used with full understanding of its interpretation and significance. The study also highlighted the importance of the influence of exogenous noise. Even the same CA value might correspond to different autoregulation levels in different ‘noise’ conditions.

## Methods

### Ethical approval

The data in this study was gathered during a retrospective analysis of data collected prospectively from 1146 head-injured patients admitted to the Addenbrooke’s Hospital Neurocritical Care Unit between 1992 and 2017^13,46^. 138 recordings from 34 randomly selected traumatic brain injured (TBI) patients with a clinical need for intracranial pressure monitoring and computerized signal recordings were included for this analysis. The anonymised computerized data storage protocol was reviewed and approved by the local ethics committee of Addenbrooke’s Hospital, Cambridge University and the neuro critical care unit User’s Group. The study was approved by the institutional ethics committee (30 REC 97/291). Inclusion criteria were: traumatic brain injury as diagnosis on admission; invasive monitoring of arterial blood pressure, monitoring of flow velocity through transcranial Doppler (TCD) for at least 30 min and mortality and GCS data available. All patients were sedated, ventilated and managed according to a CPP protocol for management of head injury with CPP maintained at> 60 mm Hg^47^.

### Data acquisition

Arterial blood pressure was monitored in the radial or femoral artery (Baxter Healthcare CA, USA; Sidcup, UK) with a zero calibration at the level of the right atrium (1992–2015) and at the foreamen of Monroe (2015–2017). Cerebral blood velocity was monitored from the middle cerebral arteries (MCA) via the transtemporal windows bilaterally using Doppler Box (DWL Compumedics, Singen, Germany) or Neuroguard (Medasonic, Fremont, CA, USA).The insonation depth was from 4 to 6 cm and the examinations were performed during the first 3 days after head injury^48^.

Between 1992 and 1996 data trends (1-minute time averages) were collected at 50 Hz with non- propriety software developed in house. From 1996–2002 Data were sampled at 100 Hz with proprietary data acquisition software and one minute trends were stored (ICM, Cambridge Enterprise, Cambridge, UK) and from 2002–2017 data were collected using ICM + , (Cambridge Enterprise, Cambridge, UK, http://icmplus.neurosurg.cam.ac.uk). Artefacts introduced by tracheal suctioning, arterial line flushing or transducer malfunction were removed manually. Data were recorded and analyzed anonymously as a part of standard audit approved by Neurocritical Care Users Group Committee.

### Data analysis

#### Generation of artificial CBFV waveforms

Artificial mean CBFV waveforms were generated according to the mathematical model proposed by Tiecks *et al*.^1^ using real ABP recordings (Supplementary S1 Equation 1–4). The model provides a second order high pass filter representation of the relationship between ABP and CBFV that can be adjusted for different ‘strengths’ of CA (graded by the autoregulation index - ARI). The strength of CA is divided into 10 levels, through a set of parameters: the time constant (tau), damping factor (D), and the autoregulatory dynamic gain (K), (Supplementary Table S1)^1,49^. Higher ARI denotes good CA, while ARI = 0 indicates completely abolished CA. Each ABP recording was used to generate ten simulated CBFVs according to those 10 levels of CA^1,50^.

#### Transfer Function phase, gain and coherence calculation

TF phase, gain and coherence between the real ABP and the generated flow velocities were calculated through Fourier Transform (FFT) algorithm^9,37,51^. Choice of parameter settings followed the recommendations of the International Cerebral Autoregulation Research Network (CARNet)^37,38^. The analysis mainly focuses on two frequency ranges: very low frequency range (VLF, 0.02~ 0.07 Hz) and low frequency range (LF, 0.07~ 0.2 Hz). A 300-s window was used to generate TF parameters, and was updated every 10 s to produce continuous TF parameters. Generated CBFV and ABP were first normalized into z scores (mean subtracted, and divided by the standard deviation), which can transform all the data into the same scale and handle outliers very well. Then the normalized CBFV and ABP were divided into four data segments of 120-second duration (amounting to 50% segment overlap) and transformed with the FFT algorithm (Welch method)^23,52^. The cross-spectra and auto-spectra of ABP and CBFV, the TF squared coherence were estimated using the average value of the four segments through the method described below^29^.

The auto- and cross-spectra of input (real ABP) and output (simulated CBFV)^9,53,54^ were calculated by averaging (denoted by the ‘expectation’ operator E) over repeated windows of the complex product of the signals using Eqs. 1 to 3. Spp and Svv represent auto-spectrum of blood pressure (P(t)) and flow velocity (V(t)) respectively. Spv(f) is the cross spectrum which represents a common variability in the two signals as a function of frequency.

1 $${\rm{Spp}}({\rm{f}})={\rm{E}}[{\rm{P}}({\rm{f}})\ast {\rm{P}}({\rm{f}})]$$

2 $${\rm{Svv}}({\rm{f}})={\rm{E}}[{\rm{V}}({\rm{f}})\ast {\rm{V}}({\rm{f}})]$$

3 $${\rm{Spv}}({\rm{f}})={\rm{E}}[{\rm{P}}({\rm{f}})\ast {\rm{V}}({\rm{f}})]$$

A 300-s moving window was used to generate continuous TF parameters. The time series were divided into 4 segments with 120 s recording each and transformed with the FFT algorithm using 50% overlap of segments (Welch method). The mean Spp, Svv and Spv of the four segments were then used for TF calculation (Eq. 4). H_R_ and H_I_ are the real part and imaginary part of H(f)^55^.

4 $${\rm{H}}({\rm{f}})=\frac{{\rm{Spv}}({\rm{f}})}{{\rm{Spp}}({\rm{f}})}$$

5 $${{\rm{H}}}_{{\rm{f}}}={{\rm{H}}}_{{\rm{R}}}+{{\rm{H}}}_{{\rm{I}}}\cdot {\rm{j}}=|{\rm{H}}({\rm{f}})|\times {e}^{j{\rm{\varphi }}({\rm{f}})}$$

j refers to imaginary unit, |H(f)| refers to TF gain and φ(f) refers to TF phase, which are descried below. The TF gain (|H(f)|) indicates the magnitude of change in CBFV that is caused by a change in ABP. The TF phase (φ(f)) describes the phase shift from input to output at a specific frequency^23,56^. They can be obtained through 6 and 7. Following reviewers’ comments and feedback, phase shift was unwrapped and limited to a range of [−180^0^,180^0^], and negative values were deleted.

6 $$|{\rm{H}}({\rm{f}})|={[{{{\rm{H}}}_{{\rm{I}}}}^{2}+{{{\rm{H}}}_{{\rm{R}}}}^{2}]}^{1/2}$$

7 $${\rm{\varphi }}({\rm{f}})={\tan }^{-1}\left[\frac{{{\rm{H}}}_{{\rm{I}}}({\rm{f}})}{{{\rm{H}}}_{{\rm{R}}}({\rm{f}})}\right]$$

We use gain_VLF for abbreviation of gain in VLF range, and gain_LF for gain in LF. Similarly, phase_VLF and phase_LF refer to phase in VLF and LF range, while coh_VLF and coh_LF stand for squared coherence in VLF and LF range respectively.

Coherence reflects the degree of the linear relationship between the complex values of input and output signals at different frequencies. The squared coherence is defined as squared modulus of the cross spectrum normalised by the product of the two autospectra (8), its value ranges from 0 to 1.

8 $${\rm{\gamma }}=\frac{\parallel {\rm{Spv}}({\rm{f}}){\parallel }^{2}}{{\rm{Spp}}({\rm{f}}){\rm{Svv}}({\rm{f}})}$$

If the output is a purely linear transformation of the input process, the numerator is identical to the denominator and so the coherence is 1 at all frequencies. Any deviation from linearity in the relationship between input and output or a presence of any exogenous ‘noise‘ will act to decrease the numerator, and so the coherence, toward zero.

### Mx calculation

Mx, time correlation coefficient between 10 s averages of ABP and the artificial mean CBFV, was calculated using a 300 s data window. Positive Mx indicates passive relationship between ABP and CBFV, while Mx close to 0 or negative implies good autoregulation.

All the parameters were averaged over all recordings for each patient for further analysis. Therefore, each patient finally only had one Mx, one TF phase_VLF, phase _LF, gain_VLF, gain _LF, Coh_VLF, Coh_LF at each ARI level^57^.

### Adding noise to the artificial data and testing the robustness of CA parameters

In order to investigate robustness of the examined parameters and their relationships in a more ‘real-life’ scenario, we simulated exogenous noise by adding colored noise to the generated CBFV signals. In brief, each generated CBFV signal was used to create Gaussian white noise at three SNR (10 $lo{g}_{10}\left(\frac{{P}_{signal}}{{P}_{noise}}\right)$) levels: −5 dB, −0.5 dB and 5 dB, representing high, medium and low noise signals. The highest SNR of 5 dB was selected according to the study by Katsogridakis *et al*.^45^. Then the Gaussian noise was filtered through a 6th-order Butterworth filter with the cut-off frequency of 0.2 Hz. The colored Gaussian noise signals were finally added to each simulated CBFV.

### Effect of noise

In order to analyze the effect of noise on the CA parameters, the standard deviation of mean Mx (or TF parameters) across all the patients at each ARI level was calculated. Thus, under each noise situation, 9 SD values of Mx (or TF parameters) were obtained corresponding to 9 ARI levels. Then the mean value of the 9 SD values was calculated at each noise level and was compared to evaluate the influence of noise on different CA parameters.

### Statistics

SPSS software (version 19, IBM, Armonk, NK, USA) was used for statistical analysis. The parameters were averaged across all the recordings for each patient before we analyzed the relationship among different CA parameters. Pearson’s correlation coefficient (r) was used to examine the linear correlation between different CA parameters. The significance of the correlation coefficient was tested using student t-test, with p < 0.05 representing statistical significance. A One Way ANOVA was used to tell whether there is significant difference among different levels of noise on standard deviation of each CA parameter. If the p value of One Way ANOVA was smaller than 0.05, an additional multiple comparison test (Bonferroni) was used to find out where the significant difference was located. A linear regression was performed to describe the relationship between Mx and ARI as well as Mx and phase.

## Supplementary information

Supplementary information.

## Data Availability

We have uploaded all the data set using in this study for the readers who want to try the method. The data can be downloaded by clicking the link below: ‘ Liu, Xiuyun (2019), Assessment of cerebral autoregulation indices – a modelling perspective, figshare, https://figshare.com/s/59f6e84c29e9479d19d4’.
