# Supplementary material for: Assessment of cerebral autoregulation indices – a modelling perspective
Source: Sci Rep. 2020 Jun 15;10:9600. doi: 10.1038/s41598-020-66346-6 (PMC7295753; doi:10.1038/s41598-020-66346-6)
Supplement: Supplementary file 1 — Supplementary information. [file 41598_2020_66346_MOESM1_ESM.pdf]

Supporting Information

Paper title: Assessment of cerebral autoregulation indices – a modelling perspective

Xiuyun Liu[1,2*], PhD, Marek Czosnyka[1,3], PhD, Joseph Donnelly[1,4], PhD, Danilo Cardim[1,5], PhD, Manuel Cabeleira[1], MSc, Despina Aphroditi Lalou[1], MD, Xiao Hu[6], PhD, Peter J. Hutchinson[1], PhD, Peter Smielewski[1], PhD

Running title: assessment of cerebral autoregulation

1. Brain Physics Laboratory, Division of Neurosurgery, Department of Clinical Neurosciences, Addenbrooke's Hospital, University of Cambridge, Cambridge, UK

2. Department of Anesthesiology & Critical Care Medicine, School of Medicine, Johns Hopkins University, Baltimore, MD, USA

3. Institute of Electronic Systems, Warsaw University of Technology, Poland

4. Department of Anaesthesiology, University of Auckland, Auckland, New Zealand

5. Department of Neurology and Neurotherapeutics, University of Texas Southwestern Medical Center, Dallas, TX, USA

6. School of Nursing, Duke University, Durham, NC, USA

S1 Tiecks' ARI Model [1]

The cerebral blood flow velocities in response to different levels of cerebral autoregulation, was evaluated by a second-order linear differential equation (Supplementary equation 1-4)[1] , where C is a constant value, denoting the initial value of ABP. ABP(n) represents mean ABP at time (sample) n and f stands for the sampling frequency of ABP. Tiecks' model separated CA into 10 levels through a set of parameters, which are the value of time constant (tau), damping factor (D), and the autoregulatory dynamic gain (K) (Supplementary Table 1). x_1 and x_2 are intermediate, state variables, which are set to zero at the start of the simulation process.

$$\mathrm{P(n) = ABP(n) - c} \qquad (1)$$

$$x_1(n) = x_1(n-1) + \frac{P(n) - x_2(n-1)}{f \cdot tau} \qquad (2)$$

$$x_2(n) = x_2(n-1) + \frac{x_1(n) - 2D \cdot x_2(n-1)}{f \cdot tau} \qquad (3)$$

$$V(n) = P(n) - K \cdot x_2(n) \qquad (4)$$

Supplementary Table 1. Parameters used in Tiecks' model related to different levels of cerebral autoregulation

tau [s]	D	K	ARI	dROR,%/s
…	0.00	0	0	0, Absent autoregulation
2.00	1.60	0.20	1	2.5
2.00	1.50	0.40	2	5.0
2.00	1.15	0.60	3	10.0
2.00	0.90	0.80	4	15.0
1.90	0.75	0.90	5	20.0, Normal autoregulation
1.60	0.65	0.94	6	30.0
1.20	0.55	0.96	7	40.0
0.87	0.52	0.97	8	60.0
0.65	0.50	0.98	9	80.0 Overly active autoregulation

tau is time constant, D is damping factor, K is autoregulatory dynamic gain, ARI is autoregulation index, dROR is dynamic rate of regulation according to Aaslid et al

References

1. Tiecks, F. P., Lam, A. M., Aaslid, R. & Newell, D. W. Comparison of Static and Dynamic Cerebral Autoregulation Measurements. *Stroke* **26,** 1014–1019 (1995).
